# COVID-19 and Preexisting Comorbidities: Risks, Synergies, and Clinical Outcomes

**DOI:** 10.3389/fimmu.2022.890517

**Published:** 2022-05-27

**Authors:** Banafsheh Bigdelou, Mohammad Reza Sepand, Sahar Najafikhoshnoo, Jorge Alfonso Tavares Negrete, Mohammed Sharaf, Jim Q. Ho, Ian Sullivan, Prashant Chauhan, Manina Etter, Tala Shekarian, Olin Liang, Gregor Hutter, Rahim Esfandiarpour, Steven Zanganeh

**Affiliations:** ^1^Department of Bioengineering, University of Massachusetts Dartmouth, Dartmouth, MA, United States; ^2^Department of Electrical Engineering, University of California, Irvine, CA, United States; ^3^Department of Biomedical Engineering, University of California, Irvine, Irvine, CA, United States; ^4^Laboratory for Integrated Nano Bio Electronics Innovation, The Henry Samueli School of Engineering, University of California, Irvine, Irvine, CA, United States; ^5^Department of Chemical and Biomolecular Engineering, New York University, New York, NY, United States; ^6^Department of Medicine, Albert Einstein College of Medicine, Bronx, NY, United States; ^7^Institute of Parasitology, Biology Centre Czech Academy of Science, Ceske Budejovice, Czech Republic; ^8^Department of Neurosurgery, University Hospital Basel, Basel, Switzerland; ^9^Division of Hematology/Oncology, Department of Medicine, Rhode Island Hospital and Warren Alpert Medical School of Brown University, Providence, RI, United States

**Keywords:** coronavirus disease 2019, COVID-19, immune responses, cancer, cardiovascular disease, diabetes, treatment implications

## Abstract

Severe acute respiratory syndrome coronavirus 2 (SARS-CoV-2) and its associated symptoms, named coronavirus disease 2019 (COVID-19), have rapidly spread worldwide, resulting in the declaration of a pandemic. When several countries began enacting quarantine and lockdown policies, the pandemic as it is now known truly began. While most patients have minimal symptoms, approximately 20% of verified subjects are suffering from serious medical consequences. Co-existing diseases, such as cardiovascular disease, cancer, diabetes, and others, have been shown to make patients more vulnerable to severe outcomes from COVID-19 by modulating host–viral interactions and immune responses, causing severe infection and mortality. In this review, we outline the putative signaling pathways at the interface of COVID-19 and several diseases, emphasizing the clinical and molecular implications of concurring diseases in COVID-19 clinical outcomes. As evidence is limited on co-existing diseases and COVID-19, most findings are preliminary, and further research is required for optimal management of patients with comorbidities.

## 1 Introduction

In late 2019, a large number of unexplained pneumonia cases appeared in the Wuhan province of China. As the number of cases started to increase exponentially, what occurred within the region became understood as the first outbreak of the coronavirus disease 2019 (COVID-19) pandemic. When several countries began enacting quarantine and lockdown policies, the pandemic as it is now known truly began. A point that became unequivocally clear during this time is that those with pre-existing conditions and the elderly were at much greater risk of contracting a severe acute respiratory syndrome coronavirus 2 (SARS-CoV-2) viral infection.

Many researchers have noted the higher mortality rate of COVID-19 infections in subjects with comorbidities such as hypertension, cardiovascular disease, obesity, diabetes, and cancer (1–6). Further predisposing conditions are autoimmune diseases, chronic kidney disease, chronic lung diseases like asthma, neurological conditions like dementia, liver diseases, solid organ transplant, chronic respiratory disease, Down syndrome, and alcohol consumption (2, 7–9). In this article, we highlight the potential interactions between COVID-19 and various diseases (**Table 1**) and discuss how such concurring diseases may result in more drastic, life-threatening conditions.

**Table 1:** The potential interactions between coexistence of different diseases and COVID-19.

Disease/organ	Impact on the immune system	Disease and COVID-19 shared features	References
Cancer	- Impaired lymphocyte function - Neutropenia - Increased risk of infection due to the immunosuppressed status - Decrease in white cell count caused by cytotoxic chemotherapy - Expansion of immunosuppressive myeloid cells *via* elevated pro-inflammatory cytokines - Dampened CD8+ T cell function, caused by extracellular vesicles released from B cells in response to chemotherapy - Activation of pro-inflammatory processes caused by major surgeries - Reduction in numbers of tumor-infiltrating natural killer (NK) cells and lymphocytes after surgeries	- Cytokine storm - IL-6 enhancement: directly correlated with the prognosis of patients with COVID-19 and also a driver of tumorigenesis and anti-apoptosis signaling, which is a key biomarker of cancer risk, diagnosis, and prognosis - Exhaustion of T lymphocytes contributes to weakened T cell activity - ICI and CAR-T cell therapies may exacerbate the COVID-19 hyperinflammatory state and increase mortality in cancer patients	(1, 2, 10–25)
Cardiovascular disease and hypertension	- Over-activated immune response could induce deterioration of cardiac function in fulminant myocarditis - Increased circulating cytokines promote inflammatory infiltration in off-target organs, especially the heart - Use of immune-related therapeutic drugs could trigger both injury directly induced by cardiac inflammation and indirect cardiac injury caused by systemic inflammation - Patients with hypertension have an increased risk for severe infection - Hypertension might cause CD8+ T cell dysfunction	- COVID-19 promotes the development of cardiovascular disorders - ACE-2 expression dysregulated - Injury to pericytes through virus infection can lead to dysfunction of capillary endothelial cells, inducing microvascular dysfunction - COVID-19 might lead to cardiac dysfunction and progression of atherosclerosis - In COVID-19 patients, hypertension delays viral clearance and exacerbates airway hyperinflammation - Monocytes can be activated by the vascular endothelium during hypertension, releasing cytokines - Development of stress-induced cardiomyopathy, cytokine-related myocardial dysfunction, and sepsis-associated cardiac dysfunction can be caused through advanced stages of COVID-19	(2, 21, 26–32)
Diabetes Mellitus	- Hyperglycemia weakens the host’s defense system, compromising lymphopaenia, granulocyte, and macrophage function - Hyperglycemia increases pulmonary vascular inflammation and permeability - T2DM shows a decrease in immune-effective T cells and increase in immune-suppressive T cells - Higher levels of serum-based biomarkers (IL-6, ESR, CRP, serum ferritin) - T1DM has a dysregulated Treg response with defects of Treg activation -T2DM has an extremely active Th17 response - A sustained increase in proinflammatory cytokines can be seen in both T1DM and T2DM	- Hypercoagulation - Endothelial dysfunction - Fibrosis - Pathogenic links between the two diseases, ranging from increased inflammation to detrimental effects on glucose homeostasis - Hyperglycemia may play a role in proliferating viruses through elevated glucose levels, affecting COVID-19 viral replication and inflammation - COVID-19 patients with diabetes showed lower levels of absolute lymphocyte count but higher neutrophil count	(2, 10, 33–40)
Obesity	-Alters the distribution and number of immune cells in adipose tissue - Decreased number of Treg cells, Th2 cells, and M2 macrophages - Increase in inflammatory cells like M1 macrophages and CD8+T cells - Increased lipid deposition in bone marrow and thymus with an excess of lipid storage in other tissues affects leukocyte population - Reduces the size of inguinal lymph nodes, which can hamper dendritic cell and fluid transport function - Increased leptin levels in obesity patients aggravate cases of acute respiratory distress syndrome - Higher levels of DDP4 inhibits improvement of insulin sensitivity, suppressing inflammatory response cytokines	- Abnormal insulin signaling pathway in obesity can relate to COVID-19 resistance and mortality - Adipose tissue has reservoir-like effects for COVID-19, where lipid droplets in tissues facilitate virus spread - Obesity patients possess longer COVID-19 symptoms due to viral shedding - Adipose tissue secreted IL-6, a marker of COVID-19 severity - Overabundance of amino acids can trigger mTOR pathway, supporting SARS-CoV-2 replication through utilization of host viral replication and subsequent inflammation.	(10, 36, 41–45)
Alcohol consumption	- Increases the risk of viral and bacterial infections depending on the pattern of alcohol exposure, whether is it acute or chronic - Chronic alcohol consumption drives disease progression of viral infections and lowers antibody response with vaccinations -Enhances viral entrances by increasing alveolar barrier permeability -Alveolar, myocardium, and CNS macrophages are open to oxidative stress - Inhibits adaptive immunity through suppression of T cell proliferation and induced T cell dysfunction. - Alcohol-induced neurovascular inflammatory responses	- Increased alveolar barrier permeability leads to the possible development of acute respiratory disease, the most common symptom of severe COVID-19 patients - Promote inflammatory immune responses and impair anti-inflammatory cytokines - Suppression of T cell function establishes a further synergistic effect with COVID-19	(8, 46–63)
Chronic kidney disease	- Elevated cytokines (IL-6 and CRP) - Oxidative stress	- Elevated ACE-2 expression	(4)
Chronic liver disease	- Major source of proteins with innate and adaptive immune responses	- Cirrhosis-associated immune dysfunction in addition may amplify COVID-19 symptoms	(1, 5)
Down syndrome	- Possess mild to moderate T and B cell lymphopenia - Marked decrease of naive lymphocytes	- Increase risk of COVID-19 through impaired mitogen-induced T cell proliferation and defects of neutrophil chemotaxis	(2, 7)
Autoimmune disease	- High infection risk	-Neutrophil extracellular trap production promotes pathogenic role -ANA, ANCA, and APL autoantibodies also present in COVID-19 patients	(3, 34, 35)
Neurodegenerative diseases	- Increased blood-brain barrier permeability	- Depression, Parkinson’s or Alzheimer’s patients are more susceptible to COVID-19 because of increased BBB permeability - Pre-activated microglia from previous immune challenges may also promote a more intense COVID-19 response	(8, 9, 33)

ACE-2, Angiotensin-converting enzyme 2; BBB, Blood-brain barrier; CNS, Central nervous system ; CRP, C-reactive protein ; CAR, Chimeric antigen receptor -T; ESR, Erythrocyte sedimentation rate ; ICI, Immune checkpoint inhibitors ; IL-6, Interleukin 6 ; NK, Natural killer ; T1DM, Type 1 diabetes mellitus ; T2DM, Type 2 diabetes mellitus.

## 2 COVID-19 and the Most Common Comorbidities

### 2.1 Diabetes Mellitus

**Figure 1:**
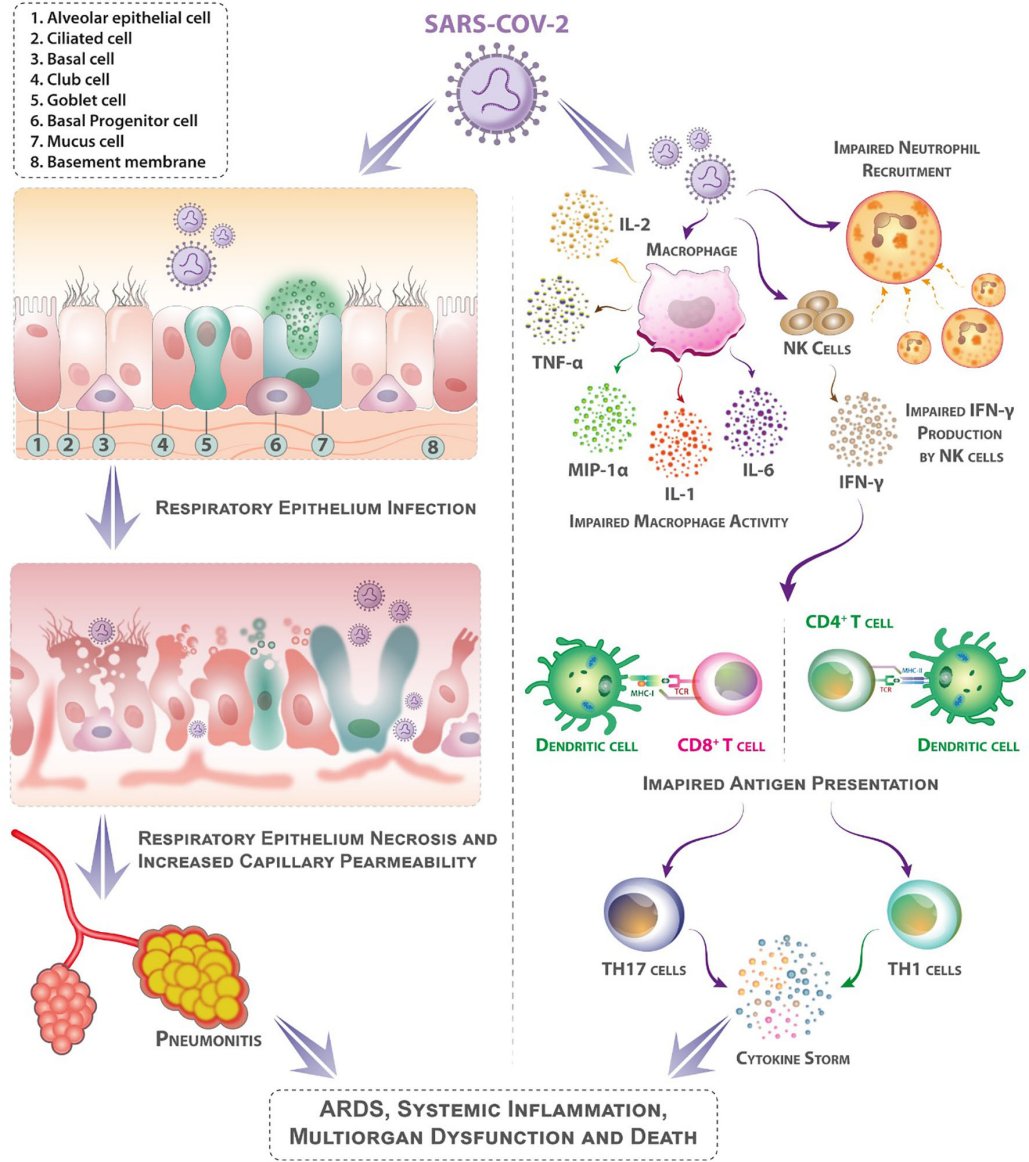
Mechanisms that may contribute to diabetes patients’ higher sensitivity to coronavirus illness (COVID-19). Following aerosolized absorption of the severe acute respiratory syndrome coronavirus 2 (SARS-CoV-2), SARS-CoV-2 infects the respiratory epithelium and other target cells by attaching to angiotensin-converting enzyme 2 (ACE-2) on their surface. Higher ACE-2 expression (as an adaptive response to elevated angiotensin-II levels) may support more efficient cell attachment and entrance into cells. Diabetes mellitus impairs early neutrophil and macrophage recruiting and function. In diabetes mellitus, a delay in the onset of adaptive immunity and dysregulation of the cytokine response can involve the onset of cytokine storm. (Patients with diabetes mellitus are likely to have suppressed antiviral IFN responses, and the delayed activity of Th1/Th17 may contribute to heightened inflammatory responses).

Diabetes occurs in two main types, type 1 and type 2, wherein those patients with type 1 diabetes produce no insulin whatsoever and those with type 2 diabetes respond to insulin inefficiently, if at all. COVID-19 severity and mortality appear linked to the existence of diabetes mellitus and individual levels of hyperglycemia (33–36). Diabetics are at higher risk of SARS-CoV-2 infection (37, 38), and poor glycemic management entails increased need for treatment and hospitalizations as well as a higher fatality rate (36, 39). Several pathophysiological processes may contribute to the higher susceptibility of diabetes mellitus patients infected with SARS-CoV-2 (**Figure 1**). Hyperglycemia, in combination with other risk factors, may modify immunological and inflammatory processes, predisposing individuals to severe, potentially fatal COVID-19. COVID-19 mortality is further increased by a multitude of related diabetic complications, such as hypertension, heart failure, obesity, and chronic kidney disease (33, 40).

#### 2.1.1 COVID-19 and Glucose Metabolism

Hyperglycemia, the condition of elevated blood glucose levels, weakens the lymphopenia, granulocyte, and macrophage functions of host defense systems (10). Blood glucose levels could thus serve as a benchmark in determining the severity of COVID-19 symptoms, as measures of fasting blood glucose have been used to indicate mortality even in those not suffering from diabetes (10). SARS-CoV-2 replication is directly increased by elevated glucose levels in human monocytes and sustained by glycolysis through the generation of mitochondrial reactive oxygen species and the hypoxia-inducible factor 1α activation (41). As a result, hyperglycemia may promote viral replication. Moreover, in animals infected with middle east respiratory syndrome coronavirus (MERS-CoV), concomitant type 2 diabetes mellitus (T2DM) resulted in an impaired immunological reaction and severe respiratory impairment (42). In rodent models of these two diseases, hyperglycemia has been shown to increase instances of pulmonary vascular inflammation and permeability, which may increase inflammatory processes related to COVID-19 (43).

In patients with compromised glucose control or diabetes mellitus, glycemic worsening is a common side effect of COVID-19. SARS-CoV infection, for example, was linked to an increase in the demand for high doses of insulin in insulin-dependent individuals (approaching or above 100 IU/day) (44). Further, variations in insulin requirements appear to be linked to inflammatory cytokine levels (44, 45). While ketoacidosis is a complication commonly related to type 1 diabetes mellitus (T1DM), it can also occur in people with T2DM suffering from COVID-19. In a systematic review, T2DM was found in 77% of COVID-19 patients who experienced ketoacidosis (46).

#### 2.1.2 Inflammation and Insulin Resistance

Insulin resistance is caused by a diminished sensitivity of tissue to insulin and corresponds to the failure of the pancreas to generate appropriate amounts of insulin for blood glucose control (47). Inflammation can be associated with an increase of insulin resistance, as it has been shown that inflammatory signals generated as a result of obesity work to activate serine kinases that, in turn, impact and block insulin action and function (48). Therefore, metabolic abnormalities, such as hypertension, obesity, and T2DM, share a common increase in adiposity caused by low-grade meta-inflammation (49). Several pathways have been proposed for virally mediated increasing insulin resistance due to inflammation (50). For example, upon infection with cytomegalovirus, glycemic control was deteriorated in prediabetic mice with hepatic insulin resistance caused by diet-induced obesity (50). Immunological imbalance and pro-inflammatory cytokines with a T helper cell type 1 signature have been shown to enhance insulin resistance in obese people (51), but their roles in COVID-19 remain unknown. In humans, acute respiratory viral infection enhances interferon gamma (IFNγ) generation and promotes muscular insulin resistance, leading to compensatory hyperinsulinemia to preserve euglycemia and promote antiviral CD8+ T cell responses (50). It is possible that such compensation fails in people with poor glucose tolerance or diabetes mellitus (52). Hyperinsulinemia can boost antiviral immunity by directly stimulating CD8+ effector T cell activity (50). As a result, throughout SARS-CoV-2 infection, the antiviral immunological and inflammatory reactions can alter sensitivity to insulin, thereby worsening glucose metabolic abnormalities. Inflammatory cells infiltrate the lungs in coronavirus-induced pneumonias like severe acute respiratory syndrome (SARS) and MERS, resulting in severe pulmonary injury, acute respiratory distress syndrome (ARDS), and/or death (53). High levels of inflammatory cells can affect the liver and skeletal muscle functions, both of which respond to insulin and absorb most of the body’s insulin-mediated glucose (54). Severe COVID-19 has also been linked to muscle weakness and elevated enzyme activity in the liver, two indicators of multiple organ failure, especially during cytokine storms (55).

There is a scarcity of information about the relation of insulin resistance and COVID-19. Nevertheless, viral attachment to angiotensin-converting enzyme 2 (ACE-2) is thought to stimulate angiotensin II (Ang II), suggesting it to be the key factor in the synergy between insulin resistance and cardiovascular disease (56, 57). ACE-2 controls blood pressure in a healthy microenvironment by transforming Ang II to Ang (1–7), consequently reducing insulin resistance and oxidative stress, and increasing GLUT4 function (58). ACE-2 expression is reduced during COVID-19 infection, resulting in increased Ang II activity, which leads to insulin resistance, oxidative stress, inflammatory responses, hypertension, and cardiac dysfunction (57). Obese and diabetic people depict higher levels of inflammation, which in turn leads to insulin resistance and vice versa (57). Inflammation is intensified during COVID-19; in case the disease coincides with obesity and diabetes, therefore, hyperinflammation, other serious conditions like lung and heart disease, or death may result (59). Elevated insulin resistance results in increased pancreatic production of ACE-2 receptors, which in turn increases affinity for the attachment of spike proteins and, ultimately, the vulnerability of patients with insulin resistance to COVID-19 infection (60). As well, the comorbidities present in insulin-resistant patients — often, hypertension, hyperglycemia, and diabetes mellitus — contribute to the severity and mortality of COVID-19 (15).

#### 2.1.3 Immunomodulation

Mechanisms connecting COVID-19 to both T1DM and T2DM have been discovered to coincide with immune function (61). As mentioned earlier, hyperglycemia can impair immune function; similarly, a dysregulated immune system has been associated with macrovascular pathology related to diabetes mellitus (62, 63). The most common pathologies observed post-mortem in patients who died from COVID-19 are diffuse alveolar destruction and inflammatory cell infiltration with significant hyaline membranes (11).

Notably, infection caused by respiratory syncytial viruses leads to the increased production of IFNγ, triggering a defense mechanism: the production of natural killer (NK) cells (12). Abundant IFNγ and activated NK cells worsen systemic inflammation in muscle and adipose tissue, reducing the body’s ability to absorb glucose (13). Moreover, in patients with poor glucose metabolism, NK cell activity is linked to impaired glucose regulation. In patients with T2DM, for example, NK cell activity is decreased compared to those with prediabetes or normal glucose tolerance (14). Furthermore, multiple regression analysis has revealed that HbA1c levels in T2DM patients can be used to predict NK cell activity (14). As a result, people with impaired glucose tolerance or diabetes mellitus have lower NK cell activity, which could clarify the sensitivity to COVID-19 and poor prognosis of diabetic patients compared to those without diabetes. Knowing the immunomodulation that occurs during COVID-19 disease is critical for determining therapeutic strategies, generating effective drugs, and understanding the disease’s pathophysiology.

A decrease in immune-effective CD4+/CD8+ TCRβ+ T cells and an increase in characteristically immune-suppressive TCRγδ+ CD4−CD8− T cells has been demonstrated in T2DM animal models; Further, the studies demonstrated a decrease in mucosa-protecting cells, showing possible effects of T2DM on the innate immune system: nasal immunity (15). Hence, patients with COVID-19 simultaneously suffering from T2DM may experience impaired function of nasal-associated lymphoid tissue (NALT) and thus olfactory dysfunction (15). Patients with T1DM have a dysregulated Treg response, with non-impaired absolute numbers of Treg cells but defects in their activation, ultimately affecting the entire regulation of immune responses. Patients with T2DM, on the other hand, depict an extremely strong Th17 response, deviating from accepted levels of Th17 activity. In Type 2 diabetes, an imbalance in Th17 and Treg cells exists, indicating a disruption in T cell homeostasis which in turn can contribute to an inflammatory state. Alterations in lipogenesis and lipolysis directly affect Th17 cell function, suggesting that, even with regulated blood glucose levels, obesity-associated T cell inflammation could be permanent. For these reasons, a sustained increase in proinflammatory cytokines exist in both T1DM and T2DM (10).

#### 2.1.4 Renin–Angiotensin–Aldosterone System

ACE-2, a component of the renin–angiotensin–aldosterone system (RAAS), has attracted much attention for its ability to act as an entrance receptor for SARS-CoV and SARS-CoV-2 (16). While its initial discover located ACE-2 expression mostly in the respiratory system (16), this has since been shown to be mistaken: immunohistochemistry has revealed only minor respiratory tract expression compared to greater expression in the intestines, kidneys, heart, vasculature, and pancreas (17). ACE-2 appears to be expressed in a variety of human cells and organs, including pancreatic islets (18).

There is evidence of a relationship between ACE-2 and glucose control. ACE-2-knockout animals were reported to be more vulnerable than wild-type animals to impairment of pancreatic β-cells under a high-fat diet (19). In addition, SARS-CoV infection can produce hyperglycemia in patients who do not have diabetes (20). This observation suggests that coronaviruses may cause islet destruction, possibly leading to hyperglycemia (20). Hyperglycemia was shown to last for three years following recovery of SARS-infection, possibly showing long-term injury to pancreatic β-cells (20). These findings suggest that ACE-2 may play a role in the relation of COVID-19 and diabetes mellitus.

### 2.2 Obesity

**Figure 2:**
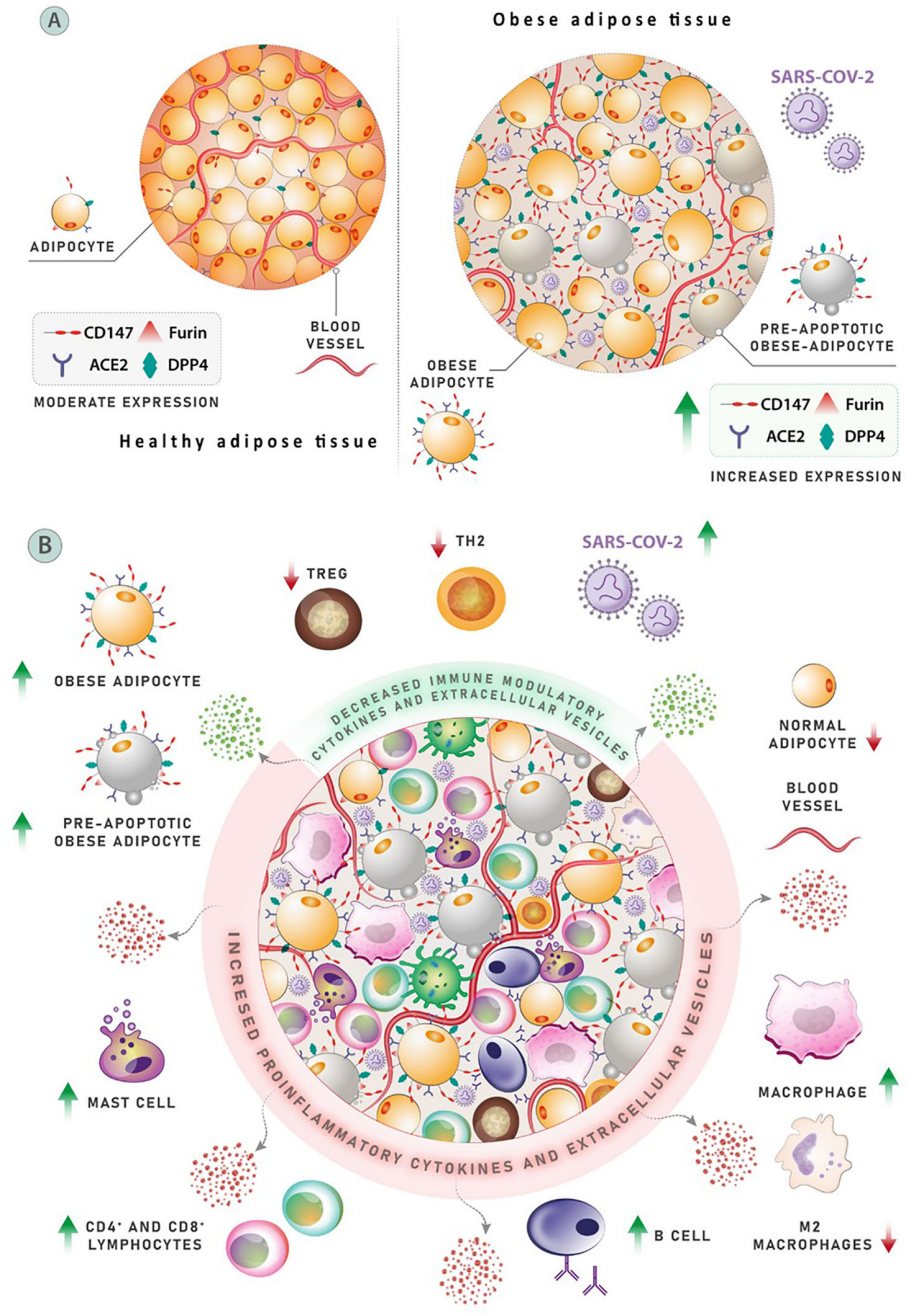
**(A)** Adipose tissue expresses the receptors ACE-2, Dipeptidyl peptidase-4 (DPP4), and CD147, as well as protease furin, following the entrance of SARS-CoV-2. The expression of these proteins is elevated in obese adipose tissues, and ACE-2 and DPP4 production in the circulation of obese people is increased. Patterns of COVID-19 morbidity and severity may be influenced by the expression of mentioned proteins, which are meaningfully associated with body mass index (BMI). **(B)** The mechanism affecting clinical outcomes and leading to poor prognosis in obese COVID-19 patients. A population of three anti-inflammatory cell types associated with proper adipose activity can be found in normal adipose tissue. Negative regulators of inflammation include T helper (Th2) cells, M-2 macrophages, and regulatory T cells (Treg). Obesity is linked to changes in the number and diversity of immune cells in the adipose tissues, including a considerable drop in Th2 cells, Treg cells, and M-2 macrophages. Conversely, the number of pro-inflammatory cells, such as CD8+ T cells and M-1 macrophages, has increased significantly. More than 40% of M-1 macrophages are found in obese, inflamed adipose tissue, which produce a variety of pro-inflammatory cytokines that cause local and systemic inflammation. Other cell types that release pro-inflammatory elements, such as neutrophils, dendritic cells, and mast cells, also contribute to inflammatory process.

For years, obesity has been a major public health issue in the United States, as it can lead to a plethora of comorbidities, and as such can be a considerable risk factor in the current pandemic climate; the condition has been reported to be an important risk factor of severe COVID-19 illness in multiple studies (21–23). With obesity, the body quickly grows adipose tissue to store extra nutrients (23). For SARS-CoV-2 entrance, adipose tissue expresses the receptors ACE-2, Dipeptidyl peptidase-4 (DPP4), and CD147, as well as the protease furin. These proteins’ expressions are elevated in obese adipose tissues, and ACE-2 and DPP4 production in the plasma of obese people is increased. COVID-19 morbidity and intensity patterns may be influenced by the expression of these proteins, which are positively associated with body mass index (BMI) (**Figure 2A**). New retrospective research found obesity to be prevalent among SARS-COV-2 cases; assessing the relation of the Body-Mass-Index (BMI) and the use of invasive mechanical ventilation (IMV), the study found that 84 (75.8%) of 124 consecutive intensive care SARS-COV-2 patients were obese (BMI > 30kg/m^2^) (24). The pattern of BMI categories in patients admitted with COVID-19 was substantially different if compared to intensive care unit (ICU) admissions the prior year for the same institution’s severe acute pulmonary disease. Compared to SARS-COV-2 patients, patients with other diseases had a lower obesity rate (25.8%) (obesity rates were equal between non-SARS-COV2 patients and the general populations of Nord and Pas de Calais). Importantly, obesity was also found to be a significant determinant in the need for intermittent mandatory ventilation (IMV). Eighty-five (68.6%) of the 124 patients required IMV, and their BMI was higher than those who did not require IMV. IMV was necessary in over 90% of individuals with a BMI greater than 35. Obesity was a substantial risk factor for severe COVID-19 in a group of patients with metabolic associated fatty liver disease (MAFLD), according to a study by Zheng et al. from three hospitals in Wenzhou, China (25). Investigators found obesity to be a strong risk factor for patients with severe COVID-19 and MAFLD. COVID-19 outcome may be influenced by increased liver fibrosis in MAFLD, according to preliminary studies (26). Further research from Rhode Island found a clear link between obesity and illness severity. The researchers looked at data from 103 adult patients who were admitted to the hospital with COVID-19. Patients with significant obesity (BMI > 35 kg/m^2^) had a higher incidence of severe COVID-19. Furthermore, obesity (BMI > 30 kg/m^2^) was found to be substantially and independently linked to the usage of IMV in COVID-19 patients (27). This hypothesis was supported by the New York University Health Center’s research on a large cohort of COVID-19 patients (n = 3615) (28). Researchers looked at BMI stratified by age in symptomatic COVID-19-positive patients who came to the hospital and discovered that patients under the age of 60 with a BMI > 30 kg/m^2^ were more than twice as likely to be admitted to the hospital and experience critical illness as those with a BMI < 30 kg/m^2^. Patients with severe obesity (BMI 35 kg/m^2^) were 3.6 times more likely to be admitted to the ICU (28). Another report from the same hospital found similar results with a larger sample size (n = 5279). The researchers found obesity to be the second-leading cause (after age) for hospitalization among COVID-19 patients (29). Research from United Kingdom linked obesity to a higher chance of mortality (30). Obesity was revealed to be a substantial risk factor for severe disease and death caused by COVID-19 in a single-center Italian study of 482 individuals. Patients with a BMI < 30 kg/m^2^ had a higher risk of severe illness, but those with a BMI > 35 kg/m^2^ had a far higher chance of death (31). Obesity predisposed young COVID-19 patients (14–45 years old) to a considerably greater mortality risk, according to a study from Zhang et al. (32). Cai et al. investigated the relationship between COVID-19 severity and obesity in a recognized hospital in Shenzhen, China, and found that patients with obesity at a higher risk of developing severe COVID-19 (64). Other nations that have been badly hit by the pandemic, such as Mexico (65), Germany (66) and Spain (67) have established a link between BMI, disease severity and death due to COVID-19. It is clear that obesity, due to the impacts it has on the proper regulation of the immune system, may be one of the most important risk factors with regards to COVID-19.

### 2.3 What Causes the Obese Person to Become so Susceptible?

Adipose tissue was long considered inactive, storing energy in lipid form in case of starvation. Now, adipose tissue is understood as a crucial endocrine organ that secretes several components (adipokines, chemokines, and cytokines) that have an important influence on metabolism and immune system function (**Figure 2B**) (68–70). Obesity is associated with significant changes in the distribution and number of immune cells in the adipose tissues, with fewer Treg cells, Th2 cells, and M2 macrophages. However, while the aforementioned cells decrease in quantity, the number of inflammation-related cells such as M1 macrophages and CD8+ T cells increases, showing a variation almost in line with autoimmune diseases (23). Relating to the previous point, obesity and related metabolic syndromes affect the proper function of lymphoid tissues and can therefore impact the spread and location of immune cells, which can then impact immune defense and T cell activity (23). Among the comorbidities associated with obesity, lipid deposition is increased in the bone marrow and thymus; an excess of lipid storage in these tissues affects the leukocyte population and can therefore affect lymphocytes and the overall functioning of immune defense (23).

As it has been shown in diabetes, insulin may prove vital to T cell metabolism and regulation. Insulin signaling results in critical immune-increasing effects on T cells, controlling their increase in number and spread while also affecting the production of cytokines and overall glucose metabolism, providing a key form of defense against possible infection. Therefore, an impacted or abnormal insulin signaling pathway can directly affect COVID-19 resistance and mortality. In addition to its previous similarities with metabolic syndrome, obesity often leads to a permanent form of insulin resistance in peripheral tissues, disrupting the insulin signaling used in this process of host defense. It seems apparent that insulin stimulation weakens signaling pathways in the lymphocytes of people with obesity or type 2 diabetes (23).

Leptin, a hormone secreted from adipocytes, helps to regulate the number of T cells, ensures their efficient functioning, and serves as a link between immune response and metabolism. Elevated levels of leptin in the bloodstream can cause an immune-impairing state best described as “leptin resistance” (23, 71, 72), and elevated levels of leptin in obese patients can cause aggravated cases of acute respiratory distress syndrome (43, 49). It is also theorized that adipose tissue has a reservoir-like effect for COVID-19; lipid droplets in these tissues may facilitate viral production and spread (23). In its function as a viral reservoir, adipose tissue can prolong virus shedding in patients with obesity (72). Due to other conditions such as an impaired immune responses and reduced macrophage activation, the prolonging of viral shedding in obese patients can be expected (72). These prolonged cases of viral shedding can also be linked to what has been described as “long COVID”, wherein symptoms associated with COVID-19 persist for much longer than the standard four-week infection period (73). The question must then be raised about whether there exists a link between pre-existing conditions such as obesity or metabolic syndrome, and the prevalence of “long COVID”.

Obesity also reduces the size of the inguinal lymph nodes, can hamper dendritic cell and fluid transport function, and therefore reduces the number of T-lymphocytes in lymph nodes (23). In addition, adipose tissue is an initial source of interleukin-6 (IL-6), an independent risk factor in determining the severity of COVID-19 in a patient. Therefore, IL-6 can be used as a biomarker for identifying severe cases (72). Hence, as a result of obesity, inflammation may increase due to the unregulated secretion of cytokines and adipokines such as IL-6, tumor necrosis factor, and C-reactive protein (CRP), resulting in the creation of a self-regenerating inflammation loop; causing the utilization of immune cells such as T cells, B cells, and macrophages; and impairing the immune system (72, 74). The unnecessary secretion of immune cells, combined with a lack of proper immune system regulation, suggests that obesity may play a large role in determining COVID-19 severity.

Obesity and an overabundance of amino acids can trigger the hyperactivation of the mTOR pathway, which in turn could support SARS-CoV-2 replication by utilizing the mechanisms involved in host viral replication and subsequent inflammation (75). Combined with the viral reservoir that can be created as a result of expanded adipose tissue, obese patients may be at a higher risk of developing severe infection. Moreover, higher levels of dipeptidyl peptidase 4 (DPP-4) in patients with obesity may additionally impair the immune system, with inhibition of DDP-4 improving insulin sensitivity in both obese and non-obese patients, thus potentially suppressing the pro-inflammatory response associated cytokines such as interleukin-10 (IL-10) and IL-6.

### 2.4 Alcohol

In most cases, the consumption of alcohol is considered detrimental to one’s health, with effects that include interference in the nervous system’s communication pathways, cardiovascular cases such as cardiomyopathy and arrhythmia, and a weakening of the immune system. Those who consume more than 20–40g/day of pure alcohol for females and 30–60 g/day for males are classified as dangerous drinkers (chronic heavy drinking or CHD) and are at higher risk of infection (76). Alcohol consumptions increase the risk of viral and bacterial infections significantly (77–79), although the severity of infection is correlated with the pattern of alcohol exposure, whether it be acute or chronic (78, 80).

An earlier investigation (into hepatitis C) clearly reported a dose-dependent relationship between viral infection and alcohol intake (81). According to a systematic study and meta-analysis (82), alcohol intake raises the risk of pneumonia due to alcohol’s effects on the immune system, raising as well the risk of malnutrition and, over time, advanced alcohol-related liver disorders (76, 82). Notably, an early investigation found a link between alcohol intake (in people who did not have an alcohol use disorder) and the level of ACE-2 in the body (particularly in the respiratory region) (83). Altogether, as recently proposed (76), harmful consumption may raise the risk of lung infection and deteriorate COVID-19 outcome, though this proposition is contradicted by the latest clinical investigation into lifestyle risk factors (84). In that cohort study, 760 people were hospitalized due to COVID-19, out of a total of 387,109 cases. Heavy alcohol consumption (measured over multiple years) was not linked to an increased risk of COVID-19 related hospitalization.

In terms of immune response, the focus should be on chronic consumption. Chronic alcohol consumption has been shown to drive disease progression in chronic viral infections such as HIV and to lower the body’s antibody response following vaccination (79). Increased alcohol intake enhances viral entrance by increasing alveolar barrier permeability and, in turn, leading to a higher risk of acute lung injury (8, 85) and the possible development of ARDS (8, 86), the most common symptom in patients with severe COVID-19 (8, 87). Heavy alcohol use disposes alveolar, myocardium, and central nervous system (CNS) macrophages to oxidative stress, reducing the efficiency of cellular responses such as phagocytosis (88, 89). During initial COVID-19 infection, the activation of macrophages initiates the inflammatory cascade, but in severe cases elevated innate immune cytokine levels lead to a so called cytokine storm, immune exhaustion, and increased mortality (90, 91). On the other hand, alcohol consumption increases the risk of COVID-19 by pro-inflammatory immune response stimulation and impairment of anti-inflammatory cytokines (78). Furthermore, COVID-19-derived neuro-inflammatory responses could cause blood–brain-barrier (BBB) disruption and among other symptoms seizures in severe cases (92), showing an overlap of alcohol-induced neurovascular inflammatory responses (93–96). Besides negatively affecting the innate immune system, alcohol negatively impacts the proliferation and function of T cells, weakening adaptive immunity and indicating further synergies with COVID-19 (97, 98). Though less than diabetes mellitus and obesity, alcohol consumption remains an associated risk factor of COVID-19, and chronic alcohol consumption should be avoided so as not to induce a severe COVID-19 case.

### 2.5 Cancer

Cancer is among the best known and most prevalent conditions that weaken the immune system. It is likely that this weakening results from an overexpression of immunosuppressive cytokines; it has been demonstrated to involve the suppression of inflammatory signals, the slowing down of dendritic cell maturation, and an increase in immunosuppresive leukocytes (99). Risk factors related to a higher incidence of COVID-19 in cancer patients could be due to the presence of chronic inflammation (100). Additionally, when lymphocytes are impaired, risk factors increase further (101, 102). Therefore, it seems to be clear that some cancer patients are constantly in a state of immunosuppression, caused either by treatments such as chemotherapy or the disease itself, and that this immunosuppression increases the overall infection risk as compared to the general population (101, 103, 104).

While cancer develops in an immunosuppressed and -compromised environment, oncologic parients are at greater risk of infection. This risk factor is further augmented by the fact that cancer treatments also increase instances of inflammatory responses. For example, chemotherapy can impact bone marrow production, resulting in decreased white blood cell count. Alternatively, surgery can increase immune response and thus the risk of infection (1). As the tumor progresses, it can cause obstuction and disrupt natural innate barriers such as mucosal tissue and the skin, significantly increasing the risk of infection in these patients in combination with the aformentioned factors resulting from treatment and the use of medical devices (102). Additionally, the prolonged use of corticosteroids, which are administered as a supportive therapy, can harm adaptive immunity and neutrophil function, increasing the risk of COVID-19 infection (1).

Chemotherapy may subject patients to certain agents that release tumor-associated macrophages in turn may increasing interleukin-10 levels which could then lead to supression of t cytotoxic T cell function due to decreased IL-12 expression. Additionally, CD8+ T cell activity may be weakened (105). Though chemotherapy is definitely an effective treatment, it can also lead to a multitude of detrimental side effects, such as disruption to the immune suppressive environment in immune and tumor cells, which can cause lymphopenia. It can also lead to the release of antigens resulting in cell death and immune suppressive cell apoptosis (10). However, it can be theorized that these immune changes in chemotherapy depend on the amount of the dose received (106). Surgery exists as an alternative or complementary treatment to chemotherapy, but neither is it without its own drawbacks, such as a decrease in lymphocytes and natural killer (NK) cells, resulting in an impaired immune system possibly causing formation of micrometastases and increase of residual tumor cells. These residual cells can then secrete cytokines, increasing instances of Treg cell and myeloid-derived suppressor cells (MDSC) recruitment (105).

In the context of the current pandemic, patients who have undergone surgery, chemotherapy, or immunotherapy (e.g. immune checkpoint inhibitors (ICI)) have been at the center of conversations about risk factors and COVID-19. Patients undergoing immunotherapy like ICI probably have better immune functions than patients who are being treated with chemotherapy (2). In modern treatments developed within the field of oncology, ICIs are targeted by immunotherapy agents against anti-programmed cell death 1 (PD-1), its ligands (PD-L1 and PDL-2), and the cytotoxic T-lymphocyte-associated antigen 4 (CTLA-4). The role of these checkpoint inhibitors is to arrest the host’s immune response. In doing so, the system may become hyperactivated, such as in a cytokine storm during COVID-19 infection, and can cause severe infection (1, 107). Since both COVID-19 infection and ICI have a downstream impact on innate immunity, and since patients with shorter treatment times have also had less time to aggravate downstream effects, it can be hypothesized that patients undergoing longer ICI treatment are more likely to develop severe COVID-19 (108). Alternatively, the existence of a high mortality risk in untreated cancer patients with COVID-19 indicates that cancer alone, without its associated treatments, may affect the immune system (109). Patients with COVID-19 also depicted elevated levels of IL-6, whereas increased IL-6 is associated with cardiac dysfunction and can further increase the risk of cardiovascular incidents such as heart failure and myocardial infraction (110). In addition to its function as an indicator of cardiovascular events, IL-6 also serves as a potent reference in its signaling pathways and pathophysiology. It has been shown that IL-6 mediates malignant changes while being the primary driver behind anti-apoptotic mechanisms, and serves as an essential biomarker in determining cancer risk, prognosis, and diagnosis (100).

Having a look on the impact of infection and cancer on T cell activity, the primary contributing factor is the exhaustion of T-lymphocytes (111, 112). With increased levels of antigens, T cells and CD8 T cells experience exhaustion, and can cause dysregulated cytokine pathways, altered metabolism, and overexpression of inhibitory receptors (112). Additionally, cancer patients infected with SARS-CoV-2 may find themselves at a higher risk of developing myocardial infarctions, septic shock, and ARDS (113). With the previously mentioned ICI cell therapies, as well as other cell-based therapies, the COVID-19 hyperinflammatory condition is aggravated with treatment, and mortality is increased due to ICI-associated pneumonitis (2, 114). Both the disease itself and its treatments leave cancer patients always at higher risk for SARS-CoV-2 infection, making them among the highest risk groups in the pandemic.

### 2.6 Cardiovascular Disease and Hypertention

The COVID-19 pandemic and its associated SARS-CoV-2 infection has also placed cardiac patients among those at most risk. The infection impacts the cardiovascular system by causing myocarditis, arrythmia, cadiogenic shock, heart failure, myocarditis, and other thromboembolic events (2). The prevelance of such events has led to the hypothesis that the disease can play a role in the development of cardiovascular disorders such as those mentioned above, along with venous thromboembolism, and acute coronary syndrome (ACS) (115). The potential effects of COVID-19 on the cardiovascular system are summarized in **Table 2**.

**Table 2:** COVID-19 cardiovascular consequences.

Manifestation	Rate	Observations
Acute cardiac injury	Average of 8–12 percent (116)	The most common reported cardiovascular problem Can be caused by any of the mechanisms listed below Direct myocardial injury Systemic inflammation Myocardial oxygen demand supply mismatch Acute coronary event Iatrogenic Significantly negative prognostic value
Acute coronary event	It hasn’t been reported, although it seems to be low.	Possible mechanisms: Inflammation/increased shear stress cause plaque rupture. Pre-existing coronary artery disease gets worse
Left ventricular systolic dysfunction	Not reported	Each of the above-mentioned causes of myocardial dysfunction can result in acute left ventricular systolic dysfunction.
Heart failure	According to one study, 52% of those who suffered heart failure while infected with COVID-19 perished, while only 12 percent survived and were discharged (117).	Acute heart failure can be caused by any of the various causes of myocardial dysfunction Acute decompensation of pre-existing stable heart failure can occur when a systemic disease increases metabolic requirements
Arrhythmia	16.7% total; 44.4% in severe disease, 8.9% in moderate cases (118)	Tachyarrhythmia and bradyarrhythmia can both happen, but their precise nature is unknown.
Potential long-term consequences	It’s too early to make a judgment.	It’s too early to determine if coronavirus illness cause significant long-term consequences. Patients recovering from a similar previous condition, Severe Acute Respiratory Syndrome, had long-term lipid and glucose metabolism and cardiovascular homeostasis abnormalities (119)

ACE-2 is a receptor that has the primary function of promoting cardiovascular health; it can also, however, multiply the damage caused by different coronaviruses. In cardiac patients, ACE-2 levels and expression are low in fibroblasts as compared to the healthy control, but high in endothelial cells and cardiomyocytes, whereas the same increase is found in patients with heart failure and aortic stenosis (120). It may seem oxymoronic that ACE-2 can contribute to healthy cardiovascular function while worsening SARS-CoV-2 infection through ACE-2 dysregulation (110). Viral infection can cause endothelial cell dysfunction resulting in microvascular dysfunction, which is correlated with pericytes injury (121). ACE-2 can also be downregulated during COVID-19, possibly aggravating atherosclerosis and causing cardiac dysfunction (115). Due to the downregulation, angiotensin II accumulates, oxidative stress increases and NADPH oxidase 2 (Nox2) is activated. Nox2 levels can be correlated with troponin elevation and instances of heart failure, presenting a possible link between Nox2 activation and cardiovascular issues as a result of COVID-19. Therefore, it can be suggested that Nox2 levels could potentially serve as biomarker for COVID-19 infection (110). ACE-2 is also downregulated in older people, which intensifies the severity of their COVID-19 infection and may explains why age is a risk factor. Studies on ACE-2 regulation within the context of COVID-19 have yielded differing conclusions, potentially as a result of the novelty of the research field. While different papers proffer completely different insights, dysregulation remains the common underlying factor.

In COVID-19 patients, increased levels of high-sensitivity cardiac troponin is an indicator for mortality and myocardial injury (110, 115). In studies of hospitalized COVID-19 patients, those with increased levels of troponin T were more likely to develop arrhythmias such as ventricular tachychardia, than those hospitalized with normal troponin T levels. Moreover, endothelial and vascular injury from COVID-19 infection increases the risk of acute coronary syndrome (ACS) and thrombus formation (115). Other abnormal biomarker levels have been observed in SARS-CoV-2 infected patients, such as phospholipase A2 group VII PLA2G7, which is caused by macrophages (122). In late-stage COVID-19, immune responses may lead to cytokine-associated myocardial dysfunction, sepsis-related cardiac dysfunction, and stress-induced cardiomyopathy (115). Other biomarkers prevalent in COVID-19 patients are increased amount of D-dimer, prolonged prothrombin time, and reduced platelet counts. As a result patients must also deal with abnormalities in coagulation, which increases the risk of thromboembolic events. The combination of this inflammatory response and the overall damage caused by COVID-19 can place certain patients at greater risk of entering a hypercoagulable state (115).

During hypertension, monocytes are activated by the vascular endothelium, causing an almost uncontrolled release of cytokines, which presents a feasible relation to COVID-19 and SARS-CoV-2 (110). Overactivation of the host’s immune response can give rise to increased inflammation and deteriorated cardiac function as a result of fulminant myocarditis. The previous release of cytokines can then cause inflammation and infiltration into unrelated organs such as the heart (123). In addition, hypertension can also cause CD8+ dysfunction (110, 124). As with cancer patients, immune-related therapeutic drugs associated with hypertension treatments, such as CAR-T cell immunotheraphy and monoclonal antibodies, can give rise to both direct cardiac and systemic inflammation (123). Therefore, patients suffering from hypertension find themselves at greater risk of severe infection as a result of both the disease and its associated treatment (2). Finally, hypertension may also trigger airway hyperinflammation and slow down viral clearance, which can further contribute to disease severity (125).

### 2.7 Other Pre-Existing Diseases

As with many others of the conditions discussed here, infection risk increases with the presence of comorbidities, such as chronic kidney and liver diseases, autoimmune diseases, and Down syndrome (126–128). Other pre-existing conditions that have been shown to exacerbate COVID-19 symptoms might have the same origins; for instance, chronic kidney disease is associated with oxidative stress and elevated expression of ACE-2 and cytokines, including IL-6 and CRP (2). In addition, the liver is a major source of proteins involved in innate and adaptive immunity, while cirrhosis-associated immune dysfunction, combined with systemic and hepatic inflammation in patients with chronic liver disease, might amplify COVID-19 symptoms (126, 129). Thus, liver injury in COVID-19 patients might be immune-mediated rather than a result of direct cytopathic damage (130).

Due to similarities of clinical manifestations, immune responses, and pathogenic mechanisms in COVID-19 and autoimmune diseases, the risk of infection in patients with autoimmune diseases is high (128, 131, 132). Neutrophil extracellular trap production (NETosis) seems to play a pathogenic role in COVID-19, similar to autoimmune diseases like lupus, antiphospholipid syndrome, and anti-cytoplasmic neutrophil antibodies (ANCA)-associated vasculitis. In addition, certain autoantibodies — like antinuclear antibodies (ANA), anti-cytoplasmic neutrophil antibodies (ANCA), and antiphospholipid (APL) antibodies, which are known to occur in many autoimmune diseases — have been detected in patients with COVID-19, contributing to our understanding of how SARS-CoV-2 might be able to induce autoimmune responses (131).

The BBB is essential in protecting the central nervous system; however, viruses can lead to BBB disruption leading to CNS inflammation (133, 134). It has been observed that BBB permiability is increased in patients with neurodegenrative diseases such as Alzheimer’s disease, depression, and Parkinson’s disease, rendering these patients much more at risk of COVID-19 (134). Additionally, if a previous immune response caused activation of microglia, these can become hyperactivated and induce an uncontrolled immune response when dealing with SARS-CoV-2 (135). Down syndrome is another condition that impacts COVID-19 risk. These patients tend to display mild or moderate B and T cell lymphopenia, with an inherently depressed level of naïve lymphocytes. Thus, Down syndrome patients are at greater risk of SARS-CoV-2 infection due to their impaired antibody response to vaccines and immunization, impaired T cell proliferation, and defective neurophil chemotaxis, among other immune system commorbidities and abnormalities (9, 127).

Obesity, diabetes, cardiovascular disease, and even factors of age and biological sex have been shown to directly influence the severity of COVID-19 symptoms and its mortality. However, the specific role of each of these conditions is not easy to distinguish. Each may interact with the others in yet unknown ways, and further assessments of different combinations of these comorbidities are necessary.

## 3 Other Factors

### 3.1 Age

What could almost be considered an inarguable fact is that older people are at greater risk of infection and more susceptible to severe infection (118, 136). Several studies have indicated that age can be considered a risk factor in COVID-19, and that older individuals remain a high-risk group during the pandemic (136–138). Immune responses can differ within age groups, as those who are older tend to have a weaker immune response and therefore are more prone to infectious diseases such as SARS-CoV-2. Older individuals tend to have less of an ability to endure inflammatory signals and an increase in pro-inflammatory cytokine production, which could potentially lead to a cytokine storm (139).

As previously mentioned, changes in ACE-2 expression can contribute to disease severity and patient mortality, and these changes often appear in older patients. As individuals get older, ACE-2 expression in the lungs increases, as shown in studies of (both male and female) patients who were not on a ventilator at the time of death (117, 136). The upregulation of ACE-2 also occurs due to anti-hypertensive treatment, a common pre-existing condition often discussed in terms of its effect on COVID-19 mortality (136). However, a multitude of diseases tend to develop with age. With increasing age, T2DM, obesity, metabolic syndrome, and cardiovascular diseases are more likely to be present and contribute to COVID-19 mortality in the elderly.

The main role of neutrophils in immune function is to help facilitate phagocytosis; as neutrophil migration becomes more inaccurate in older patients, phagocytosis function and killing activity can thus be weakened (139). Furthermore, increases in endothelial damage with age, along with associated changes in clotting, can put older individuals at high risk of COVID-19 infection (140). Aging also impacts interferon production, causing delays in type I interferon production, which in turn impairs the functioning of natural killer cells and impacts viral clearance (139). Once infected with SARS-CoV-2, the impaired interferon production might cause imbalances in M1 and M2 macrophages (139). Finally, telomere shortening, and related DNA damage can impact different kinds of CD4 T cells, such as naïve cells and memory CD4 T cells (141).

### 3.2 Sex

There has been evidence since the beginning of the pandemic that men have a higher COVID-19 fatality rate than women, possibly as a result of different concentrations of SARS-CoV-2 receptors (142). In men, more cell types readily express ACE-2, which could lead to the higher risk associated with overexpression of ACE-2. Differences in hormones between men and women may be the indicating cause of differing mortality levels. Hormonal environments in men and women, specifically relating to androgens and estrogens, have been shown to influence adaptive and innate immunity. This hormonal assistance to the immune system could be related to the suppression of lymphocyte response that in turn facilitates the immune system’s deviation from pro-inflammatory cytokine production to anti-inflammatory cytokine production (143). Estrogen has been shown to have a protective effect on the immune system. For instance, progesterone can have several anti-inflammatory effects, mainly through the inhibition of nuclear factor kappa beta and decreases in inflammatory cytokines such as but not limited to IL-12 and IL-10 (142).

Furthermore, the female sex steroid hormones also lead to greater production of interferon-α, which derives from plasmacytoid dendritic cells, further demonstrating the inherent advantages the female immune system may have over the male immune system (144, 145). Additionally, differences in mortality between men and women can also be attributed to differences in sex chromosomes. Crucially, a number of genes located on the X chromosome play a major role in immunity. So, although there should only be one activated X chromosome in females — functionally the same as in males — evidence exists of a gene imbalance that favors females and their associated immune response to infection (146, 147). It can be inferred that the increased interferon production in females is linked to both sex hormone concentration and the number of X chromosomes present (148).

It can also be observed that women can have a more efficient anti-viral immune response than men, lending them an immediate advantage in combating the virus; however when this response is prolonged, it has the potential to lead to a more severe infection (143).

In terms of specific immunity, women demonstrate more robust CD8+ T cell activity, more CD4+ T cells, and increased B cell immunoglobulin production compared to men, who in terms of relative advantages only have more CD8+ T cells (148). Generally speaking, the female immune system can be described as being much more intensive than the male immune system, as discussed above (149). Due to the strength of their immune systems, females tend to clear pathogens much faster and have higher rates of vaccine efficacy and success. However, the strength of this immune system is also what can lead to a higher prevalence of long-term inflammatory and autoimmune diseases (149). An example of this would be the female immune response to seasonal influenza vaccines, wherein antibody responses are twice as strong (119). At the same time, though, 80% of autoimmune diseases are found in women and women infected with HIV have approximately 40% less viral RNA in their blood. These examples support the previous claims that although the female immune system is more efficient at clearing out pathogens and viruses, its strength is also the reason for more prevalent autoimmune and inflammatory disorders (116, 149). 17(beta)-oestradiol (also known as E2), a biologically active form of estrogen, can increase the number of neutrophils in the blood and lungs (149). It is clear that, although not as influential as cardiovascular comorbidities, for example, sex plays a role in defining COVID-19 risk, and it appears that women may be better protected against the virus than men.

## 4 Summary and Perspectives

While respiratory impairment is the most common clinical manifestation of COVID-19, the disease’s high susceptibility and mortality in some cases points to the impact of pre-existing diseases in COVID-19 patients. Immune function deteriorates in patients with a history of cancer, diabetes, hypertension, insulin resistance, and respiratory problems, resulting in endothelial and ventilation impairment. Beyond this certainty, it must be stressed that our present understanding of how preexisting diseases affect outcomes in COVID-19 patients is insufficient. Future COVID-19 research should focus on the incidence, mechanisms, clinical manifestation, and outcomes of COVID-19 in patients who have already been diagnosed with a variety of diseases. The diagnostic and therapeutic issues arising from the coexistence of multiple diseases must also be thoroughly investigated.

## Author Contributions

BB, RE, and SZ, conceptualization and design, writing original draft. BB, MRS, MS, SN, JQH, and JT, writing – review and editing. MRS, PC, IS, and OL, figures. ME, TS, GH, RE, and SZ, conceptualization, writing, review and editing. All authors contributed to the article and approved the submitted version.

## Conflict of Interest

The authors declare that the research was conducted in the absence of any commercial or financial relationships that could be construed as a potential conflict of interest.

## Publisher’s Note

All claims expressed in this article are solely those of the authors and do not necessarily represent those of their affiliated organizations, or those of the publisher, the editors and the reviewers. Any product that may be evaluated in this article, or claim that may be made by its manufacturer, is not guaranteed or endorsed by the publisher.

## Abbreviations

ACE-2, angiotensin-converting enzyme 2; ACS, acute coronary syndrome; ANA, antinuclear antibodies; ANCA, anti-cytoplasmic neutrophil antibodies; Ang II, angiotensin II; APL, antiphospholipid; ARDS, acute respiratory distress syndrome; BBB, blood-brain-barrier; BMI, body mass index; CHD, chronic heavy drinking; CNS, central nervous system; COVID-19, coronavirus disease 2019; CRP, C-reactive protein; DPP-4,dipeptidyl peptidase 4; GM-CSF, Granulocyte-macrophage colony-stimulating factor; ICI, immune checkpoint inhibitors; ICU, intensive care unit; IFNγ, interferon gamma; IL-6, interleukin-6; IMV, invasive mechanical ventilation; MAFLD, metabolic associated fatty liver disease; MDSC, myeloid-derived suppressor cells; NALT; nasal-associated lymphoid tissue; NK, natural killer; Nox2, NADPH oxidase 2; PD-1, programmed cell death 1; RAAS, renin–angiotensin–aldosterone system; SARS-CoV2, severe acute respiratory syndrome coronavirus 2; T1DM, type 1 diabetes mellitus; T2DM, type 2 diabetes mellitus.
